# Some Particulars of the Case of Ann Ferry, with the Appearances on Dissection

**Published:** 1815-05

**Authors:** Thomas Bryant

**Affiliations:** Walworth Common, Surry.


*For the Medical and Physical Journal.*

### *Some Particulars of the Case of Ann Ferry, with the Appearances on Dissection;* by Mr. T. Bryant.

ANN FERRY, the daughter of a weaver resident in Spitalfields, became, at the age of fifteen months, the subject of hooping cough, which continued four months, then leaving her in a state of convalescence. At this period her parents observed a degree of fatuity, which had not previously been apparent. When eleven years of age, she had the power of voluntarily ejecting the contents of the stomach; this, in a few months, became accompanied with cough, dyspnœa, emaciation, &c. which continued to increase rapidly until her dissolution on the 17th of Feb. last.

*Dissection.*—Between the dura mater and tunica arachnoidea, there was at least ℥iij. of a yellow-coloured fluid. The vessels of the pia mater were particularly turgid; and a number of patches of coagulable lymph were observable on the surface of the cerebrum. The right lateral ventricle contained half an ounce of the same coloured fluid as that found between the membranes. The left lateral ventricle contained ℥ij. These were all the morbid appearances in the head.—The thorax was next examined. The lungs were found extensively tuberculated, with adhesions of the pleura pulmonalis to the pleura costalis.—The contents of the abdomen were next observed. The stomach was found to be very much contracted, with very evident increased muscularity of the pylorus. A considerable quantity of coagulable lymph on the surface of the small intestines. The pancreas remarkably enlarged and indurated, feeling like cartilage between the fingers.

The above is a correct statement of the different appearances exhibited on dissection; but the history of the case is necessarily limited, not having had the advantage of acquiring knowledge of it by attendance during the indisposition of the child, but only by inquiry, through the medium of her friends, after death.

*Walworth Common, Surry.* THOMAS BRYANT.

